# Chinese Propolis: Ultrasound‐assisted enhanced ethanolic extraction, volatile components analysis, antioxidant and antibacterial activity comparison

**DOI:** 10.1002/fsn3.1997

**Published:** 2020-11-20

**Authors:** Qingzhi Ding, Arooj Rehman Sheikh, Xiangyue Gu, Juan Li, Kaihui Xia, Nianzhen Sun, Ricardo A. Wu, Lin Luo, Yong Zhang, Haile Ma

**Affiliations:** ^1^ School of Food and Biological Engineering Jiangsu University Zhenjiang China; ^2^ Institute of Food Physical Processing Jiangsu University Zhenjiang China

**Keywords:** antibacterial activity, antioxidant activity, HS‐SPME‐GC‐MS, propolis, ultrasound‐assisted extraction

## Abstract

This study was aimed to enhance the extraction yield of propolis samples using ultrasound technology, analyze the volatile compounds, and compare the antioxidant and antimicrobial effect of propolis extracts of different areas. Four propolis samples were collected from different regions of China, namely: Linqing, Shandong Province (LSP); Yingchun, Heilongjiang Province (YHP); Changge, Henan Province (CHP); and Raohe, Heilongjiang Province (RHP). The ultrasound extracts of CHP and RHP showed a higher total phenolic content (TPC) of 201.78 ± 4.60 mgGAE/g and 166.071 ± 1.53 mgGAE/g, total flavonoid content (TFC) of 519.77 ± 29.90 and 341.227 ± 10.82 mg quercetin/g respectively, as well as high antioxidant and antibacterial activity. Conventional extraction showed 15%–20% lower yield for TPC ranging from 72.02 ± 1.99 to 155.95 ± 3.69 mg GAE/g, TFC ranges from 129.675 ± 6.82 to 412.83 ± 12.14 mg quercetin/g, with lower antibacterial activity. The antioxidant activity of propolis extracts was determined by assays of reducing power, DPPH*, FRAP*, TEAC*, hydroxyl radical scavenging activity and superoxide anion scavenging activity. Collectively, the antioxidant activities of extracts from CHP and RHP were higher than those of the other two extracts(YHP and LSP). All the extracts showed high antimicrobial activity on *Staphylococcus aureus*, *Listeria monocytogenes*, and *Bacillus subtilis*, but no effect on *Escherichia coli*. A total of 150 compounds in propolis were detected by GC/MS. Terpenes (RHP 34%, YHP 5%, LSP 18%, and CHP 12%) and alcohols (RHP 12%, YHP 13%, LSP 12%, and CHP 10%) showed the highest relative content among all other extracts.

## Graphical abstract

Novel ultrasonic technology used to enhance yield of phenolic compounds and extract enhanced antimicrobial activity.

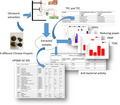

## 1 INTRODUCTION

Propolis is a material collected by bees from the sap of trees and mixed with their saliva to seal and sterilize the hive (Reis et al., 2019). Generally, propolis is composed of 50% resins, 30% vegetable balsams, 10% wax, 5% aromatic and essential oils, and 5% pollens and other substances (Rufatto et al., 2017). The chemical composition varies depending on the source of the plant. Propolis exhibits a broad spectrum of biological activities, including anticancer, antibacterial (Kasiotis et al., 2017) and antioxidant properties (Yuan et al., 2020). Many studies have indicated a particular interest in the antioxidant activities of propolis using various assays. These assays are mainly spectrophotometric, which include free radical scavenging (Pobiega et al., 2019), ferric reducing antioxidant power (Ahmed et al., 2017; Gülçin et al., 2010), and electrochemical methods (Rebiai et al., 2011). A lot of results have described the antimicrobial and antibacterial activity of propolis extracts, noting that phenolic compounds, terpenoids and nonaromatic acids are primarily responsible for the inhibiting microorganism growth (Bayram et al., 2017; Martinotti & Ranzato, 2015; Yuan et al., 2020).

The essential oils extracted from propolis exhibited antimicrobial activity mostly against gram‐positive bacteria and fungi (Franchin et al., 2016). The primary polyphenol (flavonoid) content in propolis fluctuates qualitatively and quantitatively depending on the plant's environment and ecology (Dantas Silva et al., 2017). Headspace solid‐phase microextraction (HS‐SPME) has been proposed as a dependable tool for the analysis of volatile organic compounds (Mohtar et al., 2018), because it removes many drawbacks: (a) organic extraction, (b) higher cost and (c) prolonged extraction time.

In this study, we focus on enhanced extraction of propolis compound using a novel technology to attain a higher yield at the cost of relatively less energy and time. Ultrasound wave‐mediated energized medium (solvent) indorsed easy recovery of the active compounds from the samples. Ultrasound‐assisted extraction was done from propolis to analyze the volatile compounds using HS‐SPME and GC‐MS to evaluate their antioxidant and antibacterial activities, in vitro. Results of this study prove that the proposed technology may be economically and efficiently superior for the industrial use in the manufacturing of functional foods.

## 2 MATERIAL AND METHODS

### 2.1 Materials

Gallic acid, vanillin, quercetin, ethanol, methanol, HPLC‐grade acetonitrile (ACN), acetic acid, Folin–Ciocalteu reagent, aluminum chloride, 2,2‐diphenyl‐1‐picrylhydrazyl (DPPH), and other chemicals were purchased from Sigma‐Aldrich (St. Louis, MO, USA). The purity of standard compounds was checked by HPLC–DAD analysis and was higher than 98%. All the other reagents were of analytical grade and purchased from Sinopharm Chemical Reagent Co., Ltd (Shanghai, China). *Bacillus subtilis* (No. ATCC10160), *Staphylococcus aureus* (No. ATCC25923), *Listeria monocytogenes* (No. ATCC19120) were purchased from the Microbial Strain Preservation Center, China. *Escherichia coli* (DH5α) was prepared and stored at −20ºC.

### 2.2 Sample collection, preparation, and ultrasound‐assisted extraction of propolis

#### 2.2.1 Shandong province propolis (LSP)

Samples were collected with the help of beekeepers from the area of Songlin Town, Linqing City, Shandong Province (N36°53′52.12″ E115°51′8.89″) from the plant species *Ginkgo biloba *L., *Populus alba* L, *opulus canadensis *Moench, and *Salix babylonica*. The bee species used was *Apis* *cerana* during May.

#### 2.2.2 Raohe, heilongjiang province propoliS (RHP)

Crude propolis samples were collected from Raohe city, Heilongjiang Province *(*N47°34′26″, E134°20′16). Main floras of the area include *Fraxinus mandshurica* Rupr., *Populus L*., *Quercus mongolica* Fisch, Tilia tuan Szyszyl., and Betula. Sampling was done in June and the main bee species present at that time was *Apis mellifera*.

#### 2.2.3 Changge, henan province propolis (CHP)

Samples were collected by beekeepers from the location of Nanxi Town, Changge City, Henan Province (N34°12′59.04″, E114°05′56.40″) during April and common plants there were *Paulownia fortunei*, Populus tomentosa, *opulus canadensis and* Moench, *Populus alba* L. Bee species in this area were *Apis mellifera* and *Ligustica Spinola*.

#### 2.2.4 Yingchun, heilongjiang province propolis (YHP)

Yingchun Town, Hulin City, Heilongjiang Province (N46°03′2.13″, E132°56′50.10″) was the last location for propolis sample collection during August and the bee species found there was *Apis mellifera*. Typical floras of that area were *Fraxinus mandshurica *Rupr., Phellodendron amurense Rupr, *Juglans mandshurica*, *Quercus mongolica* Fisch. Ledeb, *Ulmus pumila* L., and *Tilia tuan* Szyszyl.

#### 2.2.5 Initial processing

Propolis samples after collection were crushed using a chilled mortar at freezing temperature, then sieved using a sieve size of 40 mesh and kept at −20°C once powdered. A pulverized propolis sample (5 g) was added to 100 ml of 95% ethanol and placed in an ultrasonic device (Jiangsu Jiangda Wukesong Biological Technology Co., Ltd., Jiangsu, China) at 220 W and 40 kHz for 30 min. After three extractions, the extract was subjected to suction filtration and filtered to a volume of 300 ml to obtain the extract for further analysis. After ultrasound extraction, the mixtures were centrifuged for 10 min at 1,644 *g* (TGL‐16M High‐speed Desktop Refrigerated Centrifuge, Changsha Xiangyi Centrifuge Instrument Co., Ltd.). The supernatant was filtered using vacuum filtration and ethanol was added up to 250 ml. Supernatants of different extracts were divided into two parts: one part was prepared for detection and the other part was concentrated using a rotary evaporator at 40°C (R‐210 BUCHI Labortechnik AG, Flawil, Switzerland). Subsequently, samples were shifted to a vacuum‐drying oven to remove any residual solvent (DZF‐6050 Shanghai Yiheng Scientific Instrument Co., Ltd.). All measurements were carried out in triplicate.

### 2.3 Determination of Total Phenolic Content (TPC)

TPC was determined by the Folin–Ciocalteu assay (Hernandez Zarate et al., 2018). Samples were mixed with diluted Folin–Ciocalteu solution and 500 ml of 20% Na_2_CO_3_ solution, mixture was kept in the dark for 1 hr at room temperature and centrifuged at 872.1 *g* for 10 min, and absorption was measured at 750 nm using UV spectrophotomerer (Cary 100, Agilent Technologies, Santa Clara CA, USA). Gallic acid at various concentrations was used as a standard to construct a standard curve. TPC was denoted as mg of Gallic acid equivalent (GAE)/grams of propolis extract using a calibration curve of 50–250 mg of gallic acid/mL.

### 2.4 Determination of Total Flavonoid Content (TFC)

Total flavonoid content was determined using colorimetric method based on aluminum chloride complex formation (Hernandez Zarate et al., 2018). Briefly, 0.5 ml of 5% AlCl_3_ mixed with 2 ml of extracts in 25 ml volumetric flask distilled water was used to adjust the volume. Mixture was left in the dark at room temperature for 30 min, and absorption was measured at 425 nm using UV spectrophotometer. Quercetin was used as reference to plot a standard curve. Results were expressed as mg of quercetin of propolis extract.

### 2.5 Antioxidant assays

#### 2.5.1 DPPH scavenging assay

0.2 ml of DPPH solution (0.1 mM) was mixed with Propolis extracts at various concentrations (0–5 mg/ml) using vortex. The reference compound (Trolox) was used to draw a standard curve. Samples were then incubated in the dark for 30 min at room temperature, and absorbance was measured at 517 nm using UV‐ spectrophotometer (Wen et al., 2020). The equation used for DPPH scavenging activity was as follows:

$$\text{DPPHscavenging activity}\;(\%)=\left[1\;‐{\;\text{(Ab}}_{\text{s0}}‐{\text{Ab}}_{\text{s1}})/{\text{Ab}}_{\text{s0}}\right]\times 100$$

Where:

Abs_0_ = absorbance of the control sample;

Abs_1_ = absorbance in the presence of samples tested.

#### 2.5.2 Reducing power assay

Propolis extract was diluted in different concentrations (0–5 mg/ml) mixed with 0.1 ml of phosphate‐buffered saline (0.20 M, 6.6 pH) and 0.1 ml of 1% potassium ferricyanide [K_3_Fe(CN)_6_]. Samples were vortexed and left for incubation at 50°C for 20 min. After incubation 0.1 ml of 10% trichloroacetic acid was added to the samples to decrease the pH of the reaction medium to 4.0. Finally, 0.04 ml of 0.6 M FeCl_3_ was added and absorbance was measured at 700 nm using a UV spectrophotometer. Each sample was replicated thrice (Woźniak et al., 2019).

#### 2.5.3 Ferric reducing antioxidant power (FRAP)

All reagents were prepared according to Ding, Jiang, et al. (2019) with some modifications. FRAP reagent was prepared by adding 25 ml of 300 mM acetate buffer (pH 3.6) with 2.5 ml of 10 mM 2,4,6‐tripyridyl‐S‐triazine (TPTZ) solution (in 40 mM HCl) and 2.5 ml of 20 mM FeCl_3_.6H_2_O solution. The newly prepared reagent was heated at 37°C. The FRAP was done using the method of (Benzie & Strain, 1996). Crude extracts of propolis were dissolved in ethanol (95%) with a concentration of 50 mg/ml and diluted to 100, 80, 60, 40, 20, and 0 µg/ml. Aliquots (100 µg/ml) of newly prepared samples were mixed with 0.5 ml of FRAP reagent. Ferrous sulfate ion concentration (10–100 µM) was used as the standard, and the absorbance was measured at 593 nm. Results were expressed as µM ferrous sulfate per gram of propolis extract.

#### 2.5.4 Total Equivalent Antioxidant Capacity (TEAC)

The method used to determine the total antioxidant capacity was ABTS by consulting the method of Osés et al. (2020). Trolox at different concentrations was used as the standard. ABTS cations preparation includes oxidization of ABTS solution in water with the treatment of potassium supersulphate (molar ratio 1:0.35) and kept in the dark for 12–16 hr. Subsequently, the solution was diluted by adding 0.1 M potassium phosphate buffer (pH 7.4) to provide the absorbance of 0.70 ± 0.02 at 734 nm. The sample (200 µl) was added to 1.8 ml of the reagent, mixed and incubated at room temperature. After every 10 min, absorbance was read using a spectrophotometer. Distilled water was used as a control. Results were expressed in terms of TEAC, as μM TEAC/ g of propolis extract.

#### 2.5.5 Hydroxyl radical scavenging activity

The hydroxyl scavenging activity of samples was determined by following the method of Dai et al. (2017). A 1 ml sample (0.5–2.5 mg/ml), 0.5 ml 9 mmol/L FeSO_4,_ 1 ml 9 mmol/L salicylic acid ethanol solution, 1 ml 4.4 mmol/L H_2_O_2_ and 2 ml H_2_O were mixed at 37℃ for 60 min, followed by absorbance measurement at 510 nm (Abs_1_). Distilled water instead of the sample was used to measure the absorbance value Abs_0_, whereas distilled water without H_2_O_2_ was used to measure the absorbance value Abs_2_. The equation used for hydroxyl radical scavenging rate was as follows:

$$\text{Hydroxyl}\;\text{scavenging}\;\text{activity}\;\left(\%\right)=\left[1‐\left({\text{Abs}}_{1}‐{\text{Ab}}_{\text{s2}}\right)/{\text{Ab}}_{\text{s0}}\right]\times 100$$

#### 2.5.6 Superoxide anion scavenging activity

The hydroxyl scavenging activity of samples was determined by adopting the method of Wen et al with slight modifications. A sample solution of 0.5–2.5 mg/ml + 2.8 ml 0.1 mol/L of Tris‐HCl buffer solution at pH 8.2 was used, with double distilled water as blank. Add 0.1 ml of 3 mmol/L pyrogallol solution in water bath and start timer, preheat it at 25°C, mix it quickly to measure absorbance using spectrophtotometer at 325 nm every 30 s and finish measuring after 5 min. The antioxidant activity tests were compared to standard antioxidants such as butylatedhydroxytoluene (BHT). As the regression equation of absorbance with time, the slope is the autoxidation rate of pyrogallol. The inhibition rate of the samples to the superoxide anion was calculated as follows:

$$\text{Superoxide}\;\text{anionscavenging}\;\text{activity}\;(\%)=\left[\left(V_{0}‐V_{1}\right)/V_{0}\right]\times 100$$

Where:

*V*_0_ = The self‐oxidation rate (*△ A*/ min) of pyrogallol in the control group.

*V*_1_ = The self‐oxidation rate (*△ A*/ min) of pyrogallol in sample group.

### 2.6 Determination of the antibacterial activity of flavonoid extracted from propolis samples

#### 2.6.1 Bacterial suspension preparation

*Bacillus subtilis*, *Escherichia coli*, *Staphylococcus aureus*, and *Listeria monocytogenes* were activated in LB solid media. An inoculating ring was used to collect bacterial strains, which were then cultured for 18 hr in liquid media with continuous shaking. The concentration of the various bacterial suspensions was adjusted to 106–107 CFU/ml for further analysis (Wen et al., 2020).

#### 2.6.2 Bacterial Growth Inhibition determination assay

A sterilized spatula was used to drop and coat 120 µl of bacterial suspension uniformly. Filter paper soaked with antimicrobial propolis extract was pasted on the plate containing bacteria on agar medium. Each plate was pasted three times. Two plates were cultured in a constant temperature incubator at 37ºC for 24 hr to determine the size of the bacteriostatic zone (Kalogeropoulos et al., 2009). The size of the inhibited area was measured (mm).

### 2.7 Determination of volatile compounds by HS‐SPME GC‐MS

Analysis of the volatile compounds in propolis was done using headspace solid‐phase microextraction (HS‐SPME) combined with GC‐MS. HS‐SPME was done using a manual holder and fiber (050/30 µm DVB/CAR/PDMS, 24Ga). In a 15 ml flat bottom head vial, 0.5 g of sample was placed and sealed with a magnetic crimp cap and PTFE/silicone septa (Supelco). A thermostatic bath was used to heat the sample at 60°C for 10 min. The SPME device was introduced into the sealed vial by manually diffusing the septum and then displayed to the headspace extraction for 30 min. The SPME fiber was quickly inserted into the GC injector.

The GC‐MS analysis was performed according to the method of (Kasiotis et al., 2017) with some modifications. An Agilent 6,890 GC connected to 5973N MSD mass spectrometer framework supplied with an HP5‐MS slender section (60 m × 0.25 mm i.d., 0.32 µm film thickness) was used. Analysis of the sample was done with the column held initially at 40°C for 2 min, inclined at a rate of 10–60° and then contained for 5 min. After 2 min the temperature was straightly stepped up to 280°C with a 3°C/min heating ramp and holding at 280°C for 10 min. The carrier gas was Helium with a 1.0 ml/min flow rate. The infusion was performed in pulse splitless mode, and at injector temperature 250°C. Full scans EI (Electron Impact) spectra were recorded from 30 to 450 m/z (mass/charge) with two scans per second. The ionization voltage was 70 ev with 230°C temperature and the ionization source for EI‐ MS in positive mode.

The component identity in the extracts was referred to by the correlation of their maintenance records and mass spectra with those stored on the PC library and with those distributed in the literature. NIST05 library sources were utilized to match the identified compounds.

### 2.8 Statistical analysis

The data were expressed as the mean ± standard deviation (*SD*), analyzed by one‐way analysis of variance (ANOVA) and Tukey's test was used for comparison of the means using Minitab 18 (Minitab Inc., State College, PA, USA). Origin Pro software (2018) was used for graphical presentation. All experiments were performed in triplicate.

## 3 RESULTS AND DISCUSSION

### 3.1 Total phenolic and flavonoid contents of various extracts of propolis samples

According to the results in Table 1, TPC and TFC varied from 89.343 to 201.786 mg GAE/g of and 166.227 to 519.769 mg quercetin/g of ultrasound‐assisted extracts respectively for propolis in different areas of China with significant difference (*p* < .05). In comparison with conventional extraction, TPC varied from 72.02 ± 1.99 to 155.95 ± 3.69 mg GAE/g,TFC varied from 129.675 ± 6.82 to 412.83 ± 12.14 mg GAE/g, that is, 15%–20% lower than the ultrasound‐assisted extraction. Molnár et al. (2017) and Haile and Lin (2011) reported that TPC of propolis of different regions of Europe (Hungary and Bulgaria) and China (Zhejiang, Hubei, Hebei) varies approximately from 200 to 300 mg GAE/g, while TFC content reported is lower than that in these locations of China and TPC is higher or comparable with these values. (Kumazawa et al., 2004) reported that the TPC in propolis extract from Shandong, China was 433.8 ± 1.7 mg GAE/g, which is higher than the present study results. The reason could be due to climate change, seasonal change, and time of collection that may vary with the phenolic compounds of propolis samples.

**Table 1:** Total polyphenols and flavonoid content of different Chinese propolis extracts

Ultrasound‐assisted extracted sample	Total polyphenolic contents of propolis extracts (TPC) mg/g	Total Flavonoid content of propolis extracts (TFC) mg/g
Raohe Heilongjiang propolis( RHP)	166.07 ± 1.54	341.00 ± 10.82
Yingchun Heilongjiang propolis(YHP)	89.34 ± 1.76	166.22 ± 8.59
Linqing Shandong propolis (LSP)	154.09 ± 12.03	235.60 ± 39.70
Changge Henan propolis (CHP)	201.78 ± 4.60	519.77 ± 29.90
Conventional extraction:		
Raohe Heilongjiang propolis (CRHP)	128.56 ± 2.18	254.75 ± 6.82
Yingchun Heilongjiang propolis (CYHP)	72.02 ± 1.99	129.675 ± 5.52
Linqing Shandong propolis (CLSP)	120.43 ± 8.14	179.70 ± 10.25
Changge Henan propolis (CHP)	155.95 ± 3.69	412.83 ± 12.14

Total flavonoid content and TPC of YHP are lower as compared to those of other areas. Propolis has a wide range of phenolic compounds, mostly flavonoids (Kurek‐Górecka et al., 2013). The variations of flavonoid compounds of propolis are primarily due to the different chosen regional floras collected by honey bees. It has been found that flavonoid and other phenolic contents inhibit the growth of cancer and the development of heart disease. (Ahangari et al., 2018).

Biological activities of propolis are chiefly due to the presence of flavonoid contents in the sample (Ahangari et al., 2018). Our results have clearly shown that the difference in biological activities of samples is strictly due to the variation in flavonoid contents.

### 3.2 DPPH free radical scavenging activity of various Propolis sample extracts

**Figure 1:**
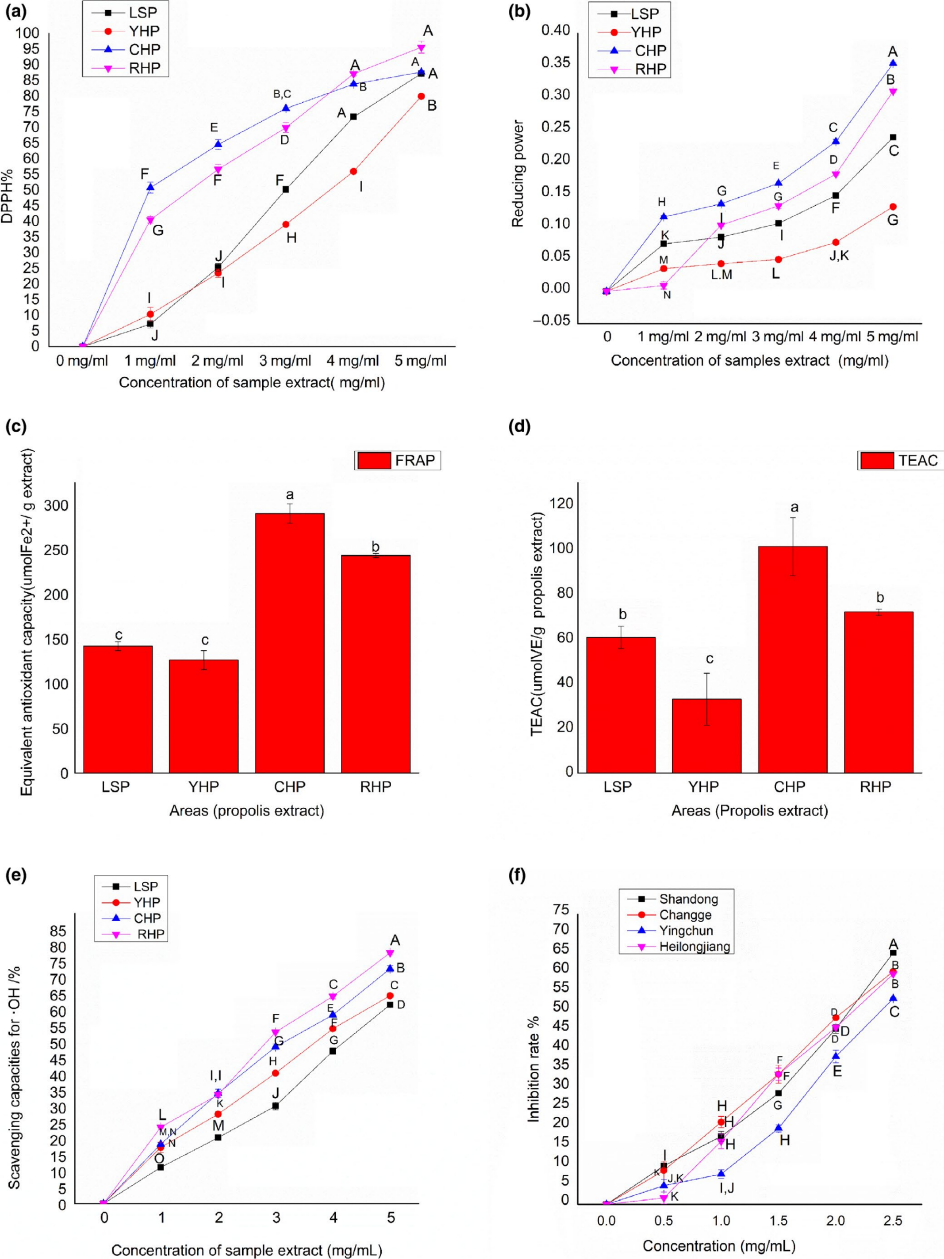
(a). DPPH activity of ultrasound‐assisted extract of propolis of different areas. Means that do not share a letter are significantly different. Figure 1(b). Reducing power rate of ultrasound‐assisted extract of propolis of different areas. Means that do not share a letter are significantly different. Figure 1(c) FRAP assay of ultrasound‐assisted extract of propolis of different areas. Means that do not share a letter are significantly different. Figure 1(d) TEAC assay of ultrasound‐assisted extract of propolis of different. Means that do not share a letter are significantly different. Figure 1(e) Hydroxyl radical scavenging activity of ultrasound‐assisted extract of propolis of different. Means that do not share a letter are significantly different. Figure 1(f) Superoxide anion scavenging activity of ultrasound‐assisted extract of propolis of different areas. Means that do not share a letter are significantly different

2,2‐diphenyl‐1‐picrylhydrazyl has been broadly used to determine the free radical scavenging activity of multiple samples (Benhanifia et al., 2013). In Figure 1(a), all extracts of propolis samples showed significant DPPH scavenging activity. Figure 1(a) shows that the order of action of DPPH free radical scavenging activity is CHP > RHP > LSP > YHP. The samples with more DPPH activity have higher phenolic contents. Phenols in the ethanolic extracts of propolis are for the antioxidant activity of propolis because of their biological properties, which is responsible in scavenging reactive oxygen species (Osés et al., 2020). YHP and LSP have higher phenolic content as compared to other samples. Phaniendra, Jestadi, and Periyasamy (2015) reported that propolis samples from China and Brazil have vigorous DPPH free radical scavenging activity ranging from 98.80 ± 1.0% to 70.50 ± 1.2%. Similar results were also published by (Hatano et al., 2012), which demonstrate that Chinese propolis has more vigorous DPPH free radical activity than Brazilian propolis.

### 3.3 Reducing power activity of different propolis extracts of samples

Various studies have shown that Chinese propolis has high reducing activity of phenolic compounds. Greek, Polish, and Slovenian propolis have high reducing power (Yang et al., 2011). In this study, propolis samples collected from different areas of China showed varying reducing power in varying dilutions. The sample with the highest reducing power activity was CHP; the sample with the lowest reducing power was YHP. The order of activity of all the samples, as shown in Figure 1(b), is CHP > RHP > LSP > YHP.

### 3.4 Ferric Reducing Antioxidant Power (FRAP) of propolis extracts

The antioxidant power of propolis samples from different regions of China was determined by using ferric, reducing antioxidant power (FRAP). Figure 1(c) shows significant differences (*p* < .05) between LSP and all other areas except for YHP samples. The propolis extracts of CHP and RHP also showed significant differences (*p* < .05) in the FRAP activity from all other areas. The FRAP values of ultrasound‐assisted extract samples ranged from 126 ± 10.60 to 290.34 ± 10.80 µmol/g ofextract. Figure 1(c) shows the FRAP activity of different samples among them CHP extracts showed the strongest FRAP activity (290.348 ± 10.80 µmol/g of extracts). The order of activity of propolis samples from different areas of China is CHP 290.35 ± 10.80 > RHP 243.35 ± 2.40 > LSP 141.93 ± 4.90 > YHP 126.47 ± 10.60. (Socha et al., 2015) previously reported the FRAP activity of extracts from Shandong, China (89.20 ± 3.80 μg/ml), Henan, China (34.5 ± 1.1) μg/mL, and different areas of Brazil ranges from 45.40 ± 2.40 μg/m to 32.10 ± 0.50 μg/mL, which is slightly lower than our results.

### 3.5 TEAC of Ultrasound‐assisted extracts of Chinese propolis from different areas

The Propolis antioxidant effect was determined by Trolox equivalent antioxidant capacity (TEAC) per gram of propolis sample. According to the results shown in Figure 1(d), all Propolis samples showed significant difference (*p* < .05) whereas, in comparison with the propolis samples of RHP and LSP was slightly insignificant (*p* < .05, *p* < 10%). The extracts of propolis extracts from CHP, RHP and YHP areas are significantly different from their mean in comparison with all the other areas. (*p* < .05). The order of activity of different samples collected is CHP > RHP > LSP > YHP, clearly shown in Figure 1(d). TEAC of propolis extracts ranged from 36.76 ± 11.6 to 106.73 ± 12.9 µmol Trolox/g of extracts. Hatano et al. (2012) reported that the TEAC values of Brazilian propolis ranged from 8,491.5 to 8,773.6 µmol TEAC g^−1^ propolis extracts. Augusto‐Obara et al. (2019) indicated that the TEAC values of Tunsanian propolis ranged from 109.76 to 252.9 μmol TEAC/g. Gargouri et al. (2019) evaluated the TEAC activity of extracted propolis samples from northeast Spain which ranged from 560–1,430 L mol Trolox/g.

### 3.6 Hydroxyl radical scavenging activity of samples extract

The extracted samples hydroxyl scavenging activity was reviewed using the Fenton reaction, and the trend is shown in Figure 1(e). The extracted samples showed significantly different scavenging activity except CHP and YHP area extracted samples with a concentration of 1 mg/ml( *p* < .01). All the samples showed the highest activity with a concentration of 5 mg/ml. Propolis samples extract from RHP showed the highest activity of 77.81%. The order of activity was RHP > CHP > YHP > LSP. Previously, Bonvehí and Gutiérrez (2011) concluded that the hydroxyl scavenging activity of Chinese and Brazilian propolis is 59.61 ± 19.92 µg·mL‐1 for Chinese propolis and 54.42 ± 10.32 µg·mL‐1 for Brazilian propolis and Chinese propolis showed a lower hydroxyl scavenging activity than Brazilian propolis.

### 3.7 Superoxide anion scavenging activity of extract of propolis of different areas

The developing of pathophysiological conditions and redox cell signalling is significantly completed by production of superoxide radical species. In most of the biological reaction medium, these species are comparatively unreactive. O2•− is also a precursor of many strong oxidants. Figure 1(f) presents the inhibition rate of ultrasound‐assisted extracts of different areas with variable concentrations. The scavenging activity was CHP > RHP > LSP > YHP. Though our data is higher, some of the previous results by de Francisco et al. (2018) studies superoxide scavenging activity of two kinds of peroplis samples from Brazil showed 48.08 ± 4.28, 138.29 mg/ml and 34.0 ± 2.0 mg/ml.

### 3.8 Antibacterial activity of different Chinese propolis extract

The ultrasound‐assisted extract of propolis was used to determine the antibacterial activity of propolis. Our previous research revealed that the antibacterial activity of Chinese propolis increased by using ultrasound to extract propolis. Moreover, the extraction of flavonoids and polyphenols yield also increased (Ding, Wu Chen, et al., 2019). The results of the antibacterial assay of selected bacterial strains (Table 2) show the highest and the lowest inhibition in the growth of these bacteria. The extracts of RHP and CHP showed the highest inhibition of *Listeria monocytogenes* than any other samples because of higher phenolic and flavonoids concentration (Table 1). LSP and YHP also showed significant effects on the inhibition of bacterial growth. Pazin et al. (2017) reported the antimicrobial activity of Turkish propolis on microorganisms like *Bacillus subtilis* (20 ± 2 mm), *Staphylococcus aureus* (18 ± 3 mm), and *Escherichia coli* (10 ± 2 mm) with the minimum inhibition concentration of 20–25 µg/ml. Moreover, Bayram et al. (2017) worked on different samples from Chile and Spain on the same bacterial strains. It is evident from the present study that gram‐positive bacteria were more sensitive to Chinese propolis extract, as shown in Table 2 *(* ***RHP*** *: Listeria monocytogenes* 21.58 ± 3.6 mm* > Staphylococcus aureus* 17.12 ± 0.91 mm* > Bacillus subtilis* 16.05 ± 0.54 mm* > E.coli*0 ***YHP*** *: Listeria monocytogenes* 18.95 ± 0.16 mm* > Staphylococcus aureus 18.27 ± 0.25 mm > Bacillus subtilis* 15.07 ± 0.11 mm *E.coli*0; **LSP** *Listeria monocytogenes* 16.77 ± 1.02 mm* > Staphylococcus aureus* 15.40 ± 0.54 mm* > Bacillus subtilis* 16.33 ± 1.03 mm* > E.coli* 0; **CHP** *Listeria monocytogenes* 17.70 ± 0.75 mm* > Staphylococcus aureus* 15.27 ± 0.16 mm* > Bacillus subtilis* 16.67 ± 0.65 mm* > E. coli*0*)*, so Chinese propolis has very little or no effect on gram‐negative bacteria (*E. coli*). In contrast extract from conventional procedure showed antimicrobial acitivity but the effect was lower than ultrasound‐assisted extracts; that is, the highest activity was from YHP: *Staphylococcus aureus* 17.59 ± 0.21 mm and *Listeria monocytogenes* 17.12 ± 0.37 mm to 0 mm for *E.coli* as both ultrasound‐assisted and conventional extraction did not show any effect on *E.coli* (Table 2). Gram‐negative bacteria have a complex chemical structure, and their flexible cell wall contains polysaccharide; these characteristics account for its toxicity in microorganisms. Another main reason for the resistance against propolis extract is the presence of the multidrug obstruction pumps, which expels the entrance of external poisons over the external layer (Tukmechi et al., 2010). Likewise, this bacterial group has a higher lipid ration than that detected in gram‐positive bacteria (Revilla et al. (2017). Mohdaly et al. (2015) also reported no effect on *E. coli* and other gram‐negative bacteria in his research using Brazilian propolis extract. However, other authors have reported propolis extract from Argentina that affects *E. coli* (Dantas Silva et al. (2017). The antibacterial mechanism of propolis is linked to some of its constituents such as higher concentration of flavonoids components like Galangin, pinocembrin and pinobanksin which possess higher antimicrobial activity (Popova et al., 2017). The reason behind the different antibacterial activity of propolis is the collection region and species of bees. Flavonoids affect the membrane of the bacteria, causing permeability alteration within the inner microorganism membrane (Przybyłek & Karpiński, 2019).

**Table 2:** Antibacterial activity of ultrasound‐assisted and conventional extracts of different Chinese Propolis

Propolis extracts (Ultrasound ‐assisted extracts)	Inhibition zone diameter (mm)			
	*Bacillus subtilis*	*Staphylococcus aureus*	*Listeria*	*E.coli*
Raohe Heilongjiang propolis (RHP)	16.05 ± 0.54	17.12 ± 0.91	21.58 ± 3.60	0
Yingchun Heilongjiang propolis (YHP)	15.07 ± 0.11	18.27 ± 0.25	18.95 ± 0.16	0
Linqing Shandong propolis( LSP)	16.33 ± 1.03	15.40 ± 0.54	16.77 ± 1.02	0
Changge Henan propolis (CHP)	16.67 ± 0.65	15.27 ± 0.16	17.70 ± 0.75	0
Conventional extraction				
Raohe Heilongjiang propolis (CRHP)	15.62 ± 0.23	15.77 ± 0.35	18.45 ± 0.24	0
Yingchun Heilongjiang propolis (CYHP)	14.28 ± 0.29	17.59 ± 0.21	17.12 ± 0.37	0
Linqing Shandong propolis(CLSP)	14.76 ± 0.20	15.02 ± 0.37	14.89 ± 0.86	0
Changge Henan propolis (CCHP)	15.25 ± 0.29	14.53 ± 0.31	15.74 ± 0.51	0

### 3.9 Analysis of volatile compounds in raw propolis by HS‐SPME GC‐MS

The chemical composition of volatile compounds depends mainly on the flora and collection site (Wojtyczka et al., 2013). Previously, Salatino, Fernandes‐Silva, Righi, and Salatino (2011) determined 99 compounds from propolis samples collected from Italy by using HS‐SPME GC‐MS. In our study, a total of 150 compounds were identified in the propolis samples collected from different regions of China.

According to the results (Table 3), LSP contains 58 kinds of substances, accounting for 38% of the total peak area. CHP comprises 51 substances, accounting for 34.0% of the total peak area. RHP contains 74 elements, accounting for 49.3% of the total peak area. YHP contains 41 kinds of substances, accounting for 27.3% of the total peak area.

**Table 3:** Volatile compounds in propolis samples determined by GC‐MS

Time/min	Flavor substances	Chemical formula	Raohe Heilongjiang (RHP) Relative content %	Yingchun Heilongjiang (YHP)	Linqing Shandong (LSP)	Changge Henan (CHP)
Acids						
23.27	Acetic acid	C_2_H_4_O_2_	2.06	1.26	–	0.73
27.79	Butyric acid	C_4_H_8_O_2_	–	–	0.14	–
32.78	Citric acid	C_5_H_8_O_2_	–	–	3.04	1.41
44.38	benzoic acid	C_7_H_6_O_2_	–	6.35	8.33	5.82
32.93	2‐Buten‐1‐ol.3‐methyl	C_5_H_8_O	1.19		–	–
	Acid(relative content%)		3.25	7.61	11.51	7.96
	Number of acid substances/one		2.00	2.00	3.00	3.00
Esters						
7.2	Ethyl Acetate	C_4_H_8_O_2_	–	0.02		
15.51	4‐ Pentene‐1‐acetate	C_7_H_12_O_2_	–	–	–	0.14
17.52	Prenyl acetate	C_7_H_12_O_2_	–	–	0.10	
17.32	2‐methyl‐2‐buten‐1‐ol ester	C_7_H_12_O_2_	–	–	0.14	–
22.93	Ethyl octanoate	C_10_H_20_O_2_	–	–	–	0.29
23.19	Methyl formate	C_2_H_4_O_2_	–	–	1.11	–
27.3	3‐methyl‐2‐buten‐1‐ol benzyl ester	C_10_H1_6_O_2_	–	–	0.93	–
30.42	Acetate	C_9_H_10_O_2_	–	1.26		
32.48	Phenylacetate	C_10_H_12_O_2_	0.68	–	–	0.67
37.32	Phenylpropanol acetate	C_9_H_12_O	0.36	0.43	–	–
47.1	Dioctyl adipate	C_22_H_42_O_4_	–	–	1.07	–
	Ester (relative content/%)		1.04	1.71	3.35	1.10
	Number of esters		2.00	4.00	4.00	3.00
Alcohols						
7.28	Ethanol	C_2_H_6_O	–		–	1.63
13.15	Ethyloctinol	C_10_H_18_O	–	0.23	–	
15.97	Prenol	C_5_H_8_O	0.42	0.32	–	
17.44	3‐methyl‐2‐buten‐1‐ol	C_5_H_10_O	–	0.43	0.65	0.53
17.68	Acid 2‐isoamyl alcohol	C_7_H_12_O_2_	0.15	–	–	
19.62	2‐methyl‐2‐buten‐1‐ol	C_5_H_10_O	0.42	0.32	0.96	0.30
19.82	2‐Buten‐1‐ol. 3‐methyl‐	C_5_H_10_O	0.53	–	–	–
21.38	2‐Isopropyl‐5‐methyl‐1‐heptanol	C_11_H_24_O	–	0.42	–	–
25.78	Linalool	C_10_H_18_O	–	–	0.89	–
29.78	Terpineol	C_10_H_18_O	0.13	–	–	1.76
33.65	Benzyl Alcohol	C_7_H_8_O	5.27	12.3	6.70	–
34.46	Phenylethanol	C_8_H_10_O	7.85	6.89	7.05	6.65
38.04	Guaiol	C_15_H_26_O	1.54	1.22	4.12	1.29
38.94	Cedarol	C_15_H_26_O	0.59	1.07	0.49	0.35
39.12	tetrahydro‐‐. à. 5‐ Trimethyl‐5‐ (4‐methyl‐3‐cyclohexene) ‐2	C_15_H_26_O_2_	–	–	0.65	0.87
39.24	Bismuth oleyl alcohol	C_15_H_26_O_2_		–	1.23	–
40.03	Agarospirol	C_15_H_26_O_2_	0.56	–	1.90	–
40.3	À‐red bisabolol	C15H26O	1.18	1.14	3.57	–
40.56	Guai‐1(10)‐en‐11‐ol	C15H26O	0.57	–	–	–
40.8	α‐Cadinol	C15H26O	–	0.29	–	–
40.89	Beta‐camphorol	C_15_H_26_O_2_	–	1.78	2.97	1.41
41.6	Cinnamyl alcohol	C_9_H_10_O	1.900.	1.02	1.18	1.89
	Alcohols (relative content/%)		19.21	26.18	31.18	13.16
	Number of alcohols		12.00	13.00	13.00	10.00
Terpenes						
10.25	Terpene	C_10_H_16_	–	–	0.12	0.06
10.41	α‐Pinene	C_10_H_16_	0.58		–	–
13.07	Bicyclo [3.1.1] heptane. 6.6‐dimethyl‐2‐methylene‐. (1S) ‐	C_10_H_16_	0.16		–	–
11.52	Terpene	C_10_H_16_	–	–	0.02	–
14.53	Myrcene	C_10_H_16_	–	–	0.02	–
15.37	Carene ‐4	C_10_H_16_	0.03	–		–
15.83	Citrus limene	C_10_H_16_	0.37		0.10	0.10
17.48	Bicyclo [3.1.0] hexan‐2‐ol. 2‐methyl‐5‐ (1‐methylethyl) ‐. (1à. 2à. 5à) ‐	C_10_H_18_O	0.17	–	–	–
18.68	Terpinolene	C_10_H_16_	0.21	–		
24.17	Long‐leafene	C_15_H_24_	–	–	–	1.11
24.47	Zelenene	C_15_H_24_		–	1.91	–
24.57	(+) – cyclodecene	C_15_H_24_	–	–	–	0.31
24.63	Cedrene	C_15_H_24_	0.63	–	–	
24.72	Tricyclo [5.4.0.0 (2.8)] undec‐9‐ene. 2.6.6.9‐tetramethyl‐	C_15_H_24_	1.23	–	0.83	–
25.84	[1S‐ ‐ octahydro‐4‐methyl‐8‐vinyl‐7‐ (1‐methylethyl) ‐1.4‐methanol‐1H‐indole	C_15_H_24_	–	–	–	0.60
25.88	Hexamethyl dewabenzene	C_12_H_18_		–	0.63	–
25.91	1.4‐Methano‐1H‐indene. octahydro‐4‐methyl‐8‐methylene‐7‐ (1‐methylethyl) ‐. [1S‐ (1à. 3aá. 4à. 7à. 7aá)] ‐	C_15_H_24_	0.58	–	–	
26.71	α‐Bergamotene	C_15_H_24_	0.25	––	–	–
26.87	Alpha‐cedarene	C_15_H_24_			0.29	–
27.13	Kuromatsuene	C_15_H_24_	13.18	–	–	–
27.37	α‐Guaiene	C_15_H_24_	0.61	–	–	–
27.2	1.2.3.4.5.6.7.8‐octahydro‐1.4‐dimethyl‐7‐ (1‐methylvinyl) indole	C_15_H_24_	–	–	0.34	0.63
27.53	Caryophyllene	C_7_H_6_O	3.88		1.35	3.35
27.61	Aristolochia	C_10_H_18_O		–	0.89	–
28	Tetracyclo [5.3.0.0 < 2.6 > 0.0 < 3.10>] deca‐4.8‐diene	C_10_H_10_	0.65	0.77	–	––
28.67	(E) ‐β‐farnesene	C_15_H_24_	0.64	–	0.54	–
29.31	Paclitaxel	C_15_H_24_		–	0.25	0.85
29.46	Cedarene	C_15_H_24_	0.63	–	1.55	0.34
29.47	α‐Caryophyllene	C_15_H_24_	0.98	–	–	–
29.63	1H‐Benzocycloheptene. 2.4a. 5.6.7.8‐hexahydro‐3.5.5.9‐tetramethyl‐. (R) ‐	C_15_H_24_	3.16	–		–
29.77	γ.‐Muurolene	C_15_H_24_	0.43	––	–	–
30.26	γ.‐Muurolene	C_15_H_24_	0.13		–	–
30.3	1.2.3.4.5.6.7.8‐octahydro‐1.4‐dimethyl‐7‐ (1‐methylvinyl) indole	C_15_H_24_		–	–	0.67
30.4	Guaia‐1(10).11‐diene	C_15_H_24_	0.56	–	–	–
30.45	(R)‐β‐Bisabolene	C_15_H_24_	0.6		–	–
30.64	Terpene	C_15_H_24_		–	0.64	0.94
30.66	Eudesma‐4 (14). 11‐diene	C_15_H_24_	0.36	–	–	–
30.74	(+) ‐ α‐ Cedarwood terpenes	C_15_H_24_	1.26	–	–	–
31.28	β‐cadinene	C_15_H_24_	0.5	–	–	–
31.34	α‐curcumene	C_15_H_22_	4.41	–	2.27	4.66
33.04	cis‐Calamenene	C_15_H_22_	0.27	–	–	–
34.69	α‐dihydropyrene	C_15_H_20_		0.47	0.90	–
34.87	α‐Calacorene	C_15_H_20_	0.31	–	–	–
36.93	3.4‐dimethoxy styrene	C_10_H_12_O_2_	0.56	–	–	–
37.23	Tricyclo [4.4.0.0 (2.7)] dec‐8‐ene‐3‐methanol. à. à. 6.8‐tetramethyl‐. stereoisomer	C_15_H_24_O	0.62		–	–
39.63	γ‐Eudesmole	C_15_H_26_O		1.07	–	–
39.86	2‐Naphthalenemethanol. 1.2.3.4.4a. 5.6.7‐octahydro‐à. à. 4a. 8‐tetramethyl‐. (2R‐cis) ‐	C_15_H_26_O	0.99	0.59	–	–
40.1	Guaiacene	C_15_H_24_	––	–	–	0.33
40.39	1.2.3.3a. 4.5.6.7‐octahydro‐à. à. 3.8‐tetramethyl‐5‐methanol	C_15_H_26_O		0.45	1.59	–
40.87	α‐Eudesmol	C_15_H_26_O	1.61	–	–	–
41.06	2‐Naphthalenemethanol. decahydro‐à. à. 4a‐trimethyl‐8‐methylene‐. [2R‐ (2à. 4aà. 8aá)] ‐	C_15_H_26_O	1.79		–	–
	Terpenes (relative content/%)		42.34	3.35	14.12	13.89
	Number of terpenes		48	5.00	18	12
Olefins						
5.11	1.4‐pentadiene	C_5_H_8_	–	–	0.01	–
15.96	3‐methyl‐2‐butene	C_5_H_8_O	–	–	0.30	0.38
16.35	Bicyclo [3.1.0] hex‐2‐ene. 4‐methyl‐1‐ (1‐methylethyl) ‐	C_10_H_16_	0.17	–	–	
25.79	2‐methyl‐5‐ (1.5‐dimethyl‐4‐hexenyl) ‐1.3‐cyclohexadiene	C_15_H_24_	–		–	–
26.96	2.6‐Dimethyl‐6‐ (4‐methyl‐3‐pentenyl) ‐2‐enebicyclo [3.1.1] hept‐2‐ene	C_15_H_24_	–		1.57	–
29.53	(R) ‐2.4a. 5.6.7.8‐hexahydro‐3.5.5.9‐tetramethyl‐1H‐benzocyclobutene	C_15_H_24_	–	–	–	2.87
30.35	‐‐1‐methyl‐4‐ (1‐methylene‐5‐methyl‐4‐hexenyl) cyclohexene	C_15_H_24_	–	–	–	0.52
	Olefins (relative content/%)		0.17	0	1.87	3.77
	Olefins (relative content/%) Number of olefins/ one		1.00		3.00	3.00
Aromatic Hyrdrocarbons						
17.67	Styrene	C_8_H_8_	0.6	0.23	0.23	0.26
18.27	P‐cymene	C_10_H_14_	0.27			
18.47	Isopropyltoluene	C_10_H_14_			–	0.01
21.27	1.3‐Cyclohexadiene. 1.5.5.6‐tetramethyl‐	C_10_H_16_	0.05			
24.33	6.7‐Dimethyl‐1.2.3.5.8.8a‐hexahydronaphthalene	C_12_H_18_			0.23	–
24.7	Cyclosativene	C_15_H_24_	0.34			
25.27	Longicyclene	C_15_H_24_	1.09			
26.9	Spiro [4.5] dec‐7‐ene. 1.8‐dimethyl‐4‐ (1‐methylethenyl) ‐. [1S‐ (1à. 4á. 5à)] ‐	C_15_H_24_	0.22			
29.22	4‐Methoxystyrene	C_10_H_18_O	0.85	0.45		
29.61	(1à. 4aá. 8aà) ‐1.2.4a. 5.8.8a‐hexahydro‐4.7‐dimethyl‐1‐ (1‐methylethyl) naphthalene	C_15_H_24_		0.62	3.30	0.25
30.24	(1à. 4aà. 8aà) ‐ 1.2.3.4.4a. 5.6.8a‐octahydro‐7‐methyl‐4‐vinyl‐1‐ (1‐methylethyl) naphthalene	C_15_H_24_			1.42	–
31.12	[1S‐ (1à. 4aá. 8aà)] ‐ 1.2.4a. 5.8.8a‐hexahydro‐4.7‐dimethyl‐1‐ (1‐methylethyl) naphthalene	C_15_H_24_		0.83	0.67	0.42
37.07	Cyclodextrin	C_15_H_24_O			0.34	–
39.4	Phenol. 4‐ethyl‐	C_8_H_10_O		2.25		
39.63	(2R‐cis) ‐1.2.3.4.4a. 5.6.7‐octahydro‐à. à. 4a. 8‐tetramethyl‐2‐methanol	C_15_H_26_O			1.57	0.52
40.55	Hydroxy‐5‐methylacetophenone	C_9_H_10_O_2_		0.83		
40.69	[2R‐ (2à. 4aà. 8aá)] ‐ 1.2.3.4.4a. 5.6.8a‐octahydro‐à. à. 4a. 8‐tetramethyl‐2‐methanol	C_15_H_26_O		1.51	2.62	1.27
	Aromatic hydrocarbons (relative content/%) Amount of aromatic hydrocarbons/ one		3.42	6.72	10.38	2.73
	Aromatic hydrocarbons (relative content/%) Amount of aromatic hydrocarbons/ one		7.00	7.00	8.00	6.00
Aldehydes						
12.07	Hexanal	C_6_H_12_O			0.09	0.03
12.53	Trans‐2‐methyl‐2‐butenal	C_5_H_8_O	0.09	0.18	0.19	0.14
21.83	Furfural	C_9_H_18_O			0.57	0.21
23.78	Furfural	C_5_H_4_O_2_			–	0.04
21.99	Nonanal	C_9_H_18_O	0.46			
25.54	Benzaldehyde	C_7_H_6_O	1.65	2.56	1.35	1.34
28.13	BETA‐cyclic citral	C_10_H_16_O			0.76	–
29.57	2‐hydroxybenzaldehyde	C_15_H_24_			–	–
37	Benzaldehyde. 4‐methoxy‐	C_8_H_8_O_2_		0.43		
37.33	Cinnamaldehyde	C_9_H_8_O	0.69	0.64	–	0.68
48.09	Vanillin	C_8_H_8_O_3_	–	1.21		
50.19	Benzyl Benzoate	C_14_H_12_O_2_	–	1.14		
	Aldehydes (relative content/%)		2.89	6.16	2.96	2.44
	Number of aldehydes/ piece		4.00	6.00	5.00	6.00
Others						
4.99	Hexane	C_6_H_14_		0.01	0.03	–
5.02	Acetone	C_3_H_6_O			–	0.08
8.47	2.2.4.6.6‐pentamethylheptane	C_12_H_26_		0.02	0.03	0.10
9.49	Decane	C_10_H_22_		0.02		
12.42	3.3‐dimethylhexane	C_8_H_18_			0.01	‐
16.38	Octane	C_10_H_18_O	0.45	0.23	0.86	0.63
18.94	Tridecane	C_13_H_28_	0.15			
20.2	Methylheptenone	C_8_H_14_O	0.08		0.08	0.06
21.77	N‐tetradecane	C_14_H_30_	0.11		–	–
23.52	à‐Methyl‐à‐ [4‐methyl‐3‐pentenyl] oxiranemethanol	C_10_H_18_O_2_	0.15			
27.96	10.10‐dimethyl ‐2.6‐divinyl‐bicyclo [7.2.0] undecane	C_15_H_24_			–	0.14
28.73	[1R‐ (1R *. 4Z. 9S *)] ‐ 4.11.11‐Third 8‐vinyl‐bicyclo [7.2.0] undecane	C_15_H_24_			–	0.63
36.3	phenol	C_6_H_6_O	0.13			
36.5	Benzene. 1.2‐dimethoxy‐4‐ (1‐propenyl) ‐	C_11_H_14_O_2_	0.1			
36.78	Phenol. 4‐ethyl‐2‐methoxy‐	C_9_H_12_0_2_		5.29		
39.73	4‐octadecylmorpholine	C_22_H_45_NO			–	–
40.72	Hydroxy‐2‐methylacetophenone	C_9_H_10_O_2_	0.55			
41.14	Isoannyl epoxide	C_15_H_24_O			–	1.43
43.34	2.4.6‐tri‐tert‐butylphenol	C_18_H_30_O			–	–
43.82	1‐ (4‐hydroxy‐3.5‐dibutylphenyl) ‐2‐methyl‐3‐morphinolinone	C_22_H_35_NO_3_			–	–
44.48	N. N'‐benzoic acid‐hexenediamide	C_21_H_22_N_2_O_6_	1.90		–	–
	Other categories (relative content/%)		3.62	5.57	1.01	3.07

For acid substances, LSP contains the highest content of acid materials, accounting for 11.51% of the total peak area. CHP, RHP, and YHP have 7.96%, 3.25%, and 6.35% acid relative contents, respectively. The variability in acid materials may be related to regional and climatic differences and the plants growing in each region.

Linqing, Shandong Province contains the highest content of esters, accounting for 3.25% of the total peak area, followed by YHP, where the content of esters accounted for 1.81% of the total peak area. CHP and RHP have 1.1% and 1.04% of relative ester contents of peak area, respectively. Although the content of the esters is relatively low, they are essential for the flavor, thus they were analyzed under the same detection conditions to obtain variable results.

Alcohol is the main contributors to propolis flavors. The content of alcohol in propolis in different regions is relatively high. Contents determined were 32.36%, 16.68%, 19.21%, and 27.43%, respectively, with no significant geographic variations. The terpenes ratio in RHP was the highest, accounting for 42.34%. CHP and LSP contained 3.35%, 13.95% and 14.24% terpenes relative content, respectively.

A very low content of olefins was found in the samples. The relative content of olefins in CHP was 3.77% of the total peak area. The relative content of olefins in LSP was 1.88%.

The aromatic substances in propolis contain bactericidal and bacteriostatic effects. The relative proportion of aromatic substances in LSP, CHP, RHP, and YHP accounted for 10.38%, 2.73%, 6.72%, and 3.42% of the total peak area, respectively. Aldehydes are also one of the active compounds in propolis, but the proportion and type are relatively small. The aldehydes in LSP, CHP, RHP, and YHP accounted for 2.96%, 2.44%, 2.89%, and 6.16%, respectively.

## 4 CONCLUSION

It can be concluded from the explained results that the polyphenolic compounds in propolis chiefly vary according to their botanical origin and environmental ecology of the plant. Ultrasound‐assisted extraction can increase the yield of TFC and TPC and thus may strongly enhance the antimicrobial activity of these extracts. Chinese propolis has high antioxidant and antimicrobial activity; variability in antioxidant activity of Chinese propolis is strictly related to variability in different amounts and kinds of phenolic compounds. Chinese propolis also contains much higher flavonoids than the other chemical substances such as alcohols, terpenes, aromatic acids, ketones, hydrocarbons, aliphatic acids, and their esters, thus they are very active as antioxidant agents. Chinese propolis may be used in natural antibacterial agents when extracted by ultrasound‐assisted extraction, as it is time‐saving and increases the extraction yield. Further studies about its uses and functional efficacy are warranted.

## CONFLICT OF INTEREST

There are no conflicts of interest.

## ETHICAL APPROVAL

This study does not involve any human or animal testing.
